# Administration’s Share of Personnel in Veterans Health Administration and Private Sector Care

**DOI:** 10.1001/jamanetworkopen.2023.52104

**Published:** 2024-01-18

**Authors:** Steffie Woolhandler, Andrew Toporek, Jian Gao, Eileen Moran, Andrew Wilper, David U. Himmelstein

**Affiliations:** 1 School of Urban Public Health, City University of New York at Hunter College, New York, New York; 2 Department of Medicine, Albert Einstein College of Medicine, Bronx, New York; 3 Harvard Medical School, Boston, Massachusetts; 4 Office of Productivity, Efficiency, and Staffing, Quality and Patient Safety, Office of Analytics and Performance Integration, Department of Veterans Affairs; 5 Office of the Chief of Staff, Boise Veterans Affairs Medical Center, Boise, Idaho; 6 Department of Medicine, University of Washington School of Medicine, Seattle

## Abstract

**Importance:**

Health care administrative overhead is greater in the US than some other nations but has not been assessed in the Veterans Health Administration (VHA).

**Objective:**

To compare administrative staffing patterns in the VHA and private (non-VHA) sectors.

**Design, Setting, and Participants:**

This cross-sectional study was conducted using US employment data from 2019, prior to pandemic-related disruptions in health care staffing, and was carried out between January 14 and August 10, 2023. A nationally representative sample of federal and nonfederal personnel in hospitals and ambulatory care settings from the American Community Survey (ACS), all employees reported in VHA personnel records, and personnel in health insurance carriers and brokers tabulated by the Bureau of Labor Statistics (BLS) were analyzed.

**Exposure:**

VHA vs private sector health care employment, including 397 occupations grouped into 18 categories.

**Main Outcome and Measure:**

The proportion of staff working in administrative occupations.

**Results:**

Among 3 239 553 persons surveyed in the ACS, 122 315 individuals (weighted population, 12 501 185 individuals) were civilians working in hospitals or ambulatory care; of the weighted population, 12 156 988 individuals (mean age, 42.6 years [95% CI, 42.5-42.7 years]; 76.2% [95% CI, 75.9%-76.5%] females) were private sector personnel and 344 197 individuals (mean age, 46.2 years [95% CI, 45.7-46.7 years]; 63.8% [95% CI, 61.8%-65.8%] females) were federal employees. In clinical settings, administrative occupations accounted for 23.4% (95% CI, 23.1%-23.8%) of private sector vs 19.8% (95% CI, 18.1%-21.4%) of VHA personnel. After including 1 000 800 employees at private sector health insurers and brokers and 13 956 VHA Central Office personnel with administrative occupations, administration accounted for 3 851 374 of 13 157 788 private sector employees (29.3%) vs 77 500 of 343 721 VHA employees (22.5%). Physicians represented approximately 7% of personnel in the VHA (7.2% [95% CI, 6.1%-8.2%]) and private sector (6.5% [95% CI, 6.3%-6.7%]), while the VHA deployed more registered nurses (23.7% [95% CI, 21.6%-25.8%] vs 21.2% [95% CI, 20.9%-21.5%]) and social service personnel (6.3% [95% CI, 5.4%-7.1%] vs 4.9% [95% CI, 4.7%-5.0%]) than the private sector.

**Conclusions and Relevance:**

In this study, administrative occupations accounted for a smaller share of personnel in the VHA compared with private sector care, a difference possibly attributable to the VHA’s simpler financing system. These findings suggest that if staffing patterns in the private sector mirrored those of the VHA, nearly 900 000 fewer administrative staff might be needed.

## Key Points

**Question:**

What is administration’s share of the workforce in the Veterans Health Administration (VHA) and in private sector health care?

**Findings:**

In this cross-sectional study of 122 315 health care personnel using nationally representative Census Bureau and VHA data, administrative occupations accounted for 22.5% of VHA employees vs 29.3% of private sector health workers.

**Meaning:**

These findings suggest that if health care employment patterns in the private sector mirrored those in the VHA, nearly 900 000 fewer administrative staff might be needed.

## Teaser

This cross-sectional study compares the share of administrative personnel in the Veterans Health Administration with that of the US private sector health care system.

## Introduction

Many studies have documented US health care’s high administrative costs, with several studies pointing to complex billing processes as drivers of those costs.^1,2,3,4,5,6,7,8,9,10,11^ Several analyses have compared US administrative costs or staffing with those in other nations,^12,13,14,15,16,17,18^ but to our knowledge, none have examined Veterans Health Administration (VHA) facilities.

The VHA’s 171 medical centers and 1113 outpatient sites care for approximately 9 million enrolled veterans. Those facilities are mostly financed through lump sum budgets tied to patients served and mean complexity rather than payments for individual services or patients. The VHA’s simpler funding model may streamline administration, but rigid government regulations may offset such efficiencies. We analyzed VHA and private sector health care employment to assess differences in administrative staffing levels.

## Methods

This cross-sectional study used no identifiable patient information and was therefore exempt from institutional review board review and informed consent under Department of Veterans Affairs (VA) Title 38 §16.101(b) (4). The study followed the Strengthening the Reporting of Observational Studies in Epidemiology (STROBE) reporting guideline for cross-sectional studies.

The VHA is both a health care delivery system and payer. It evaluates veteran eligibility for services, finances care at its own facilities, and devotes approximately 20% of its budget to reimbursing private sector health care institutions. Hence, our comparison encompasses personnel in clinical settings, as well as at the VHA Central Office and private sector health insurance carriers and brokers.

### Data Sources

Our analyses used 3 data sources: American Community Survey (ACS) data to assess employment in clinical settings in the private sector and VHA, US Bureau of Labor Statistics (BLS) data to assess private sector employment in health insurance carriers and brokers, and VHA personnel records to assess employment in the VHA Central Office (and for a sensitivity analysis). Specifically, we analyzed the Census Bureau 2019 ACS (obtained from the Integrated Public Use Microdata Series^19^), BLS Employment Matrix,^20^ and VHA Personnel Accounting Integrated Data (PAID) system.^21^

The ACS obtains information on a nationally representative sample of approximately 3.2 million US residents, including their demographic characteristics, income, industry of employment, occupation, and sector of employer (eg, federal government).^22^ The BLS Employment Matrix estimates employment for detailed industry groups based on the Current Employment Statistics survey of approximately 145 000 public and private employers, Occupational Employment Statistics survey, and Current Population Survey.^23^ The PAID system provides information separately for personnel employed in the VHA Central Office and other VHA sites on full-time equivalent (FTE) employment in each occupation.

### Study Variables

#### ACS Data on Personnel in Clinical Settings

In the ACS, we identified non–active-duty military persons reporting current employment in hospitals or outpatient care settings (Census industry codes 8190, 7970, 7980, 7990, 8070, and 8090), hereafter *health sector workers*. To ensure that comparisons between VHA and non-VHA personnel encompassed similar services, we excluded workers in nursing homes, other residential care facilities, and home care given that the VHA purchases most long-term care services from non-VHA agencies and facilities. Workers in those industries represented 22 017 of 366 214 VHA (6.0%) and 4 373 003 of 16 529 991 non-VHA (26.5%) health workers.

We determined worker demographic characteristics (including self-identified race and ethnicity) and annual hours of work (hours per week × weeks worked per year) based on their self-report, and for some analyses, we calculated FTE workers as individuals working 2000 hours/y. Race and ethnicity data were collected using the RACHSING variable from the Integrated Public Use Microdata Series (IPUMS) version of ACS data. ACS queries race and ethnicity in separate questions, with race categories including American Indian and Alaska Native, Asian and Pacific Islander, Black and African American, and White. Individuals with Hispanic origin are classified as Hispanic regardless of race response, and race categories are used for non-Hispanic individuals. We included race and ethnicity data because some readers may be interested in differences in the race and ethnicity of workers in the 2 sectors; to our knowledge, these data have not been reported elsewhere. The ACS classifies individuals’ self-reported occupations according to 2018 Census Occupation Codes. We grouped 397 individual occupations reported by health sector workers into 18 categories (eTable 1 in Supplement 1), 3 of them administrative: managers and related occupations; administrative support, financial; and administrative support except financial. We classified personnel as federal (hereafter *VHA*) or private sector, including self-employed individuals and those working for private employers or state or local governments.

#### BLS Data on Personnel Employed in Health Insurance Firms

To identify health insurance workers in the non-VHA sector (including the Centers for Medicare & Medicaid Services and other public insurers that pay non-VHA health care institutions), we used BLS data on “Direct health and medical insurance carriers” (North American Industry Classification System code 524114; hereafter *health insurers*) and “Insurance agencies, brokerages, and other insurance-related activities” (code 524200; hereafter *brokers*). To estimate employment in health-related brokers, we calculated the share of employees in all types of insurers (code 5241) employed by health insurers (code 524114) and applied that proportion (0.3587) to employment figures for brokers.

#### PAID System Data on VHA Personnel

Because the PAID system classifies occupation using the US Office of Personnel Management scheme^24^ rather than Census Occupation Codes used in the ACS, we reclassified PAID-reported occupations using a crosswalk from the Equal Employment Opportunity Commission (EEOC).^25^ We modified the EEOC crosswalk for obvious misclassifications (eg, the crosswalk coded practical nurses to the census occupation other technologists and technicians, which we instead coded as licensed practical nurses) (eMethods in Supplement 1). We applied the crosswalk to personnel in the VHA Central Office in our main analysis and to personnel in the Central Office and clinical sites in a supplementary analysis. The VHA Central Office provides system-wide support and oversight regarding financial and policy issues, information technology, contracting, data reporting, quality management, the Civilian Health and Medical Program of the Department of Veterans Affairs and Care in the Community, and veteran readjustment counseling programs. It also oversees patient account centers and the VHA outpatient mail-order pharmacy.

### Statistical Analysis

#### Administration’s Share of Personnel in Clinical Settings

The ACS uses the same methods to ascertain and code occupations for VHA and private sector workers. Because PAID ascertainment and coding of occupation differ from those available in the ACS, comparisons with the private sector workforce using those data are less secure. Moreover, PAID does not allow identification of long-term care workers, who were excluded from our analyses as stated previously. Hence, our main analyses of administration’s share of personnel in clinical settings relied on ACS data. For these analyses, we tabulated the share of all workers in each occupation group and summed the 3 administrative categories.

#### Administration’s Share of Personnel, Including Health Insurers and VHA Central Office

We estimated administration’s share of the total private sector health workforce by summing numbers of administrative personnel in private sector clinical settings, health insurers, and health insurance brokers and dividing by the total number of clinical setting plus insurance setting personnel. For our main analysis of administration’s share of the entire VHA workforce, we summed the number of administrative personnel employed in the VHA Central Office (derived by applying the crosswalked coding scheme to Central Office employees) and the number of VHA administrative personnel at clinical sites derived from our ACS-based analysis, adjusted for the small overestimation of VHA workers in the ACS (eMethods in Supplement 1). We then divided this sum by the total number of VHA personnel reported in the PAID system. For a supplementary estimate using PAID data only, we applied the crosswalked coding scheme to all VHA employees (ie, those working in clinical settings and the Central Office).

#### Additional Supplementary Analyses

Our ACS-based tabulation of federal health workers includes some non-VHA personnel, mostly Indian Health Service (IHS) employees, which likely caused the overcount of VHA personnel described previously. Because of this, we repeated our main analysis after excluding Arizona, New Mexico, Oklahoma, and South Dakota, where 75.9% of federal IHS employees worked in 2019.^26^ An additional supplementary analysis excluded state and local government employees. Finally, we carried out an analysis using ACS data to calculate FTE workers rather than the number of personnel.

We analyzed ACS data using SAS statistical software version 9.4 (SAS Institute) survey procedures, with Census Bureau–provided weights that adjusted for the complex sample design and nonresponse and yielded national estimates. We used replicate weights and the jackknife method (coefficient = 0.05) to calculate CIs. Because the PAID system provides data for a 100% sample, no CIs were calculated. For its tabulations, the BLS provides neither CIs nor information allowing their calculation. Data were analyzed from January 14 to August 10, 2023.

## Results

The 2019 ACS provided data on 3 239 553 persons, including 122 315 non–active-duty-military personnel in hospitals and ambulatory care (weighted population, 12 501 185 individuals). Of them, 12 156 988 individuals were private sector employees (mean age, 42.6 years [95% CI, 42.5-42.7 years]; 76.2% [95% CI, 75.9%-76.5%] females; 8.5% [95% CI, 8.3%-8.7%] Asian, 12.8% [95% CI, 12.5%-13.1%] Black, 13.1% [95% CI, 12.8%-13.3%] Hispanic, and 65.2% [95% CI, 65.0%-65.5%] White) and 344 197 individuals were VHA employees (mean age, 46.2 years [95% CI, 45.7-46.7 years]; 63.8% [95% CI, 61.8%-65.8%] females; 10.0% [95% CI, 8.9%-11.0%] Asian, 22.3% [95% CI, 20.4%-24.2%] Black, 10.3% [95% CI, 8.9%-11.8%] Hispanic, and 54.5% [95% CI, 52.6%-56.5%] White). Private sector employees included 11 508 179 individuals who worked for nongovernment employers and 648 809 individuals who worked for state and local governments. Table 1 displays characteristics of VHA and private sector personnel in clinical settings. More VHA employees reported their race and ethnicity as non-Hispanic Black, while fewer identified as Hispanic or non-Hispanic White. Compared with private sector health workers, VHA employees were older and fewer were female or had below-poverty family incomes. As expected, the unadjusted ACS-based estimate of federal employment (which includes some non-VHA federal health sector workers) was higher than the PAID figure of 321 643 FTE workers in clinical settings (ie, excluding the Central Office) in 2019.

**Table 1.** Characteristics of Study Population

Characteristic	Personnel, estimated % (95% CI) (weighted N = 12 501 185)	
	VHA (weighted n = 344 197)	Private sector (weighted n = 12 156 988)
Race and ethnicity		
American Indian or Alaska Native, non-Hispanic	2.9 (2.2-3.6)	0.4 (0.4-0.4)
Asian, non-Hispanic	10.0 (8.9-11.0)	8.5 (8.3-8.7)
Black, non-Hispanic	22.3 (20.4-24.2)	12.8 (12.5-13.1)
Hispanic	10.3 (8.9-11.8)	13.1 (12.8-13.3)
White, non-Hispanic	54.5 (52.6-56.5)	65.2 (65.0-65.5)
Sex		
Female	63.8 (61.8-65.8)	76.2 (75.9-76.5)
Male	36.2 (34.2- 38.2)	23.8 (23.5-24.1)
Age, mean	46.2 (45.7-46.7)	42.6 (42.5-42.7)
Region		
New England	3.5 (2.7-4.3)	5.3 (5.2-5.5)
Middle Atlantic	9.3 (7.9-10.7)	14.0 (14.8-14.3)
East North Central	12.0 (10.2-13.7)	15.5 (15.2-15.7)
West North Central	7.9 (6.7-9.0)	7.3 (7.1-7.5)
South Atlantic	22.2 (20.3-24.1)	19.4 (19.1-19.7)
East South Central	7.2 (6.0-8.3)	5.9 (5.7-6.1)
West South Central	12.3 (10.7-13.8)	10.7 (10.5-11.0)
Mountain	11.1 (9.6-12.5)	7.0 (6.8-7.1)
Pacific	14.7 (13.2-16.2)	14.9 (14.7-15.1)
Family income below poverty level	1.8 (1.3-2.3)	2.7 (2.6-2.9)

Abbreviation: VHA, Veterans Health Administration.

Table 2 provides proportions of VHA and private sector health workers in clinical settings by 18 occupation groups. The 3 administrative occupation groups together accounted for 2 850 574 private sector personnel (23.4% [95% CI, 23.1%-23.8%]) vs 68 000 VHA employees (19.8% [95% CI, 18.1%-21.4%]). Physicians accounted for approximately 7% of personnel in the VHA (7.2% [95% CI, 6.1%-8.2%]) and private sector (6.5% [95% CI, 6.3%-6.7%]), and information technology workers accounted for approximately 2% of personnel in the 2 sectors. However, more VHA workers were registered nurses (23.7% [95% CI, 21.6%-25.8%] vs 21.2% [95% CI, 20.9%-21.5%]) or social service workers (6.3% [95% CI, 5.4%-7.1%] vs 4.9% [95% CI, 4.7%-5.0%]), and fewer were health aides (9.8% [95% CI, 8.6%-11.0%] vs 13.4% [95% CI, 13.1%-13.6%]).

**Table 2.** Employment by Occupation Group

Occupation group	Personnel, % (95% CI) (weighted N = 12 501 185)	
	VHA (weighted n = 344 197)	Private sector (weighted n = 12 156 988)
Managers	10.1 (8.7-11.6)	8.1 (7.9-8.3)
Administrative support		
Nonfinancial	7.9 (6.7-9.1)	12.2 (12.0-12.5)
Financial	1.7 (1.1-2.3)	3.1 (3.0-3.2)
Total administration	19.8 (18.1-21.4)	23.4 (23.1-23.8)
Professional and technical except health (eg, engineers)	1.3 (0.9-1.8)	0.8 (0.7-0.9)
Social services	6.3 (5.4-7.1)	4.9 (4.7-5.0)
Physicians	7.2 (6.1-8.2)	6.5 (6.3-6.7)
Other health diagnosing (eg, dentists)	1.3 (0.7-1.8)	2.4 (2.3-2.5)
Registered nurses	23.7 (21.6-25.8)	21.2 (20.9-21.5)
Therapists	2.5 (2.0-3.1)	4.7 (4.5-4.9)
Other health assessing and treating (eg, pharmacists)	4.4 (3.7-5.2)	2.2 (2.1-2.2)
Health technologists and technicians	9.3 (8.1-10.4)	10.4 (10.2-10.7)
Licensed practical nurses	3.2 (2.4-3.9)	2.3 (2.2-2.4)
Other health services (eg, aides)	9.8 (8.6-11.0)	13.4 (13.1-13.6)
Food services	1.3 (0.8-1.8)	0.8 (0.8-0.9)
Cleaning, building services, and laundry	2.8 (2.0-3.5)	2.1 (2.0-2.2)
Building construction and maintenance	2.2 (1.7-2.8)	0.7 (0.7-0.8)
Information technology	1.6 (1.2-2.1)	1.6 (1.5-1.7)
Occupations not elsewhere classified	3.4 (2.7-4.1)	2.5 (2.5-2.8)

Abbreviation: VHA, Veterans Health Administration.

Non-VHA health insurers employed 573 300 persons in 2019. Additionally, an estimated 427 500 persons (among 1 191 700 workers in insurance brokers or agencies handling all types of insurance [35.87%]) worked on health insurance, yielding a total of 1 000 800 non-VHA health insurance personnel (eTable 2 in Supplement 1). Of 22 078 FTE VHA Central Office personnel, 13 956 individuals (63.2%) were in administrative occupations.

In the private sector, there were 3 851 374 administrative personnel among 13 157 788 workers from clinical and health insurance settings combined, accounting for 29.3% of the workforce (Table 3). The corresponding numbers for the VHA (ie, including administrative workers in clinical settings and the Central Office) were 77 500 of 343 721 VHA employees (22.5%), a difference of 6.8 percentage points less than the proportion of private sector workers (30.2% less).

**Table 3.** Administration’s Share of Total Health Care Employment

Occupation	Personnel, No.	
	VHA	Private sector
Administrative personnel		
Clinical settings^a^	63 544	2 850 574
Health insurance settings or VHA Central Office	13 956^b^	1 000 800^c^
Total	77 500	3 851 374
Total personnel, all occupations	343 721	13 157 788
Administration’s share of total employment, %	22.5	29.3

Abbreviation: VHA, Veterans Health Administration.

^a^ Source: authors’ analysis of the 2019 American Community Survey.

^b^ Source: authors’ analysis of VHA Personnel Accounting Integrated Data system data.

^c^ Source: Bureau of Labor Statistics Employment Matrix.

Using PAID system data (with occupational crosswalk) in supplementary analyses, we estimated that 70 510 of 321 643 FTE workers (21.9%) in VHA clinical settings (ie, excluding the Central Office) were attributable to administration (eTable 3 in Supplement 1). Using crosswalked PAID system data to classify all workers in the Central Office and in clinical settings yielded an estimate that 84 633 of 343 721 VHA personnel (24.6%) were administrative. ACS-based analyses using calculated FTE workers rather than numbers of personnel or excluding 4 states that account for 75% of IHS employees yielded similar results to those of our main analysis, as did analyses excluding state and local government employees (eTables 4-6 in Supplement 1).

## Discussion

This cross-sectional study found that a larger share of private sector compared with VHA personnel (29.3% vs 22.5%) worked in administrative occupations, an absolute difference of 6.8 percentage points and a relative difference of 30.2%. Nearly 4 million people were employed in health care administration in the private sector (excluding long-term care) in 2019, a number that would have decreased by nearly 900 000 individuals had staffing patterns followed those of the VHA.

Many studies have documented high administrative burdens and costs in US health care^1,2,3,4,5,6,7,8,9,10,11^ and lower burdens and costs in other nations, especially those with single-payer financing.^12,13,14,15,16,17,18^ Although most previous studies have quantified administrative burdens in terms of cost, our private sector personnel–based estimate aligns with cost-based estimates.^17^ A personnel-based McKinsey report using BLS data estimated that nonclinical support staff numbered 6.4 million in 2017, an increase of more than 1 million individuals since 2001.^27^ McKinsey figures include administrative personnel in long-term care and some workers whom we would not classify as administrative. However, no previous studies have compared administration in the VHA and private sectors in the US, to our knowledge.

The VHA’s leaner administrative staffing likely reflects the agency’s simpler financing scheme. High administrative costs in the private sector have been attributed to the complexity of collecting revenues.^28,29^ Health care institutions bill for individual patients and services and interact with insurers that have varying fee schedules, deductibles, prior-approval requirements, formularies, and referral networks. One academic health system employs 1500 FTE billing staff, sited in a 125 000-square-foot stand-alone building.^7^ Even capitation payment schemes generally entail risk-adjustment calculations requiring detailed tracking of each patient’s service use, diagnoses, and costs. In this context, investments in administration may make financial sense, for example, increasing revenue by documenting more diagnoses. Financial success is key to private sector institution growth and even survival, and executive compensation is often linked to financial metrics. This could incentivize efforts to improve efficiency but could also encourage revenue-enhancing administrative activities that add little clinical value (eg, marketing and upcoding).

In contrast, VHA hospitals and clinics are funded mostly through lump-sum budgets. The VHA does not bill Medicare or Medicaid and collects only 2.7% of its revenues from private insurers and 0.3% from patients,^30^ minimizing the need to attribute costs and charges to individual patients. In the VHA, all facilities are in network, 1 formulary applies to all patients, and few services require prior authorization. While managers must adhere to budgetary constraints and provide a volume of clinical services commensurate with their budgets, incentives based on financial metrics are minimal for hospital leaders and their institutions.

The difference in administration’s share of personnel in the VHA vs the US private sector that we found (6.8 percentage points) appears smaller than administrative cost differences between the US and single-payer nations; for example, there was a 13.2 percentage point disparity in hospital administrative costs and a 17.4 percentage point difference in system-wide administrative costs between the US and Canada.^17^ Several factors may account for the VHA’s less streamlined administration; unlike in Canada, where all residents are automatically and equally eligible for care, the VHA must determine eligibility based on veteran disability level, veteran income, and other factors. Additionally, the VHA serves as an insurer for some non-VHA care, spending approximately 20% of its budget purchasing private sector care.^30^

Our findings address the relative administrative efficiency of the VHA and private sectors but do not speak to differences in overall costs. A 2014 Congressional Budget Office review^31^ found suggestive but not conclusive evidence that VHA care was cheaper than private sector care. A 2022 quasiexperimental study^32^ of patients with myocardial infarction who had both Medicare and VHA coverage found 21% lower costs and lower mortality in the VHA. This finding was consonant with an analysis that found fewer complications among patients who underwent knee replacement in the VHA.^33^ A 2023 comprehensive review^34^ concluded that VHA care was generally of equal or better quality compared with the private sector.

### Limitations

This study has several limitations. The ACS does not distinguish VHA employees from other federal health sector workers, such as 13 601^26^ and 1873^35^ federal employees of the IHS and the National Institutes of Health Clinical Center, respectively. However, excluding 4 states where most IHS employees work did not affect our findings, and the number of personnel in other non-VHA federal health institutions is likely too small to skew our results.

Our ACS-based figures excluded personnel in long-term care settings, where administration’s share of costs is particularly high,^17^ as well as health-related administrative personnel sited at revenue cycle–management firms (which employed more than 60 000 people in 2022^36^), nonmedical parent firms (eg, private equity), software vendors, or consulting firms. Given that many such workers would likely have administrative occupations, we undercounted those occupational categories in VHA and private sector settings.

Our analysis did not account for the time clinicians devote to administration and financial matters, which is likely greater in the private sector. Our estimate of administrative personnel in the VHA likely includes some workers devoted to arranging private sector care rather than administering clinical services within the VHA.

Additionally, our VHA staffing figures encompass some VHA personnel working in VHA housing, education, vocational training, and other nonmedical programs, where administration’s share of employment is probably larger than the mean. However, our VHA estimates excluded private sector insurance personnel, who play a role in administering VHA payments for private sector care. In the private sector and VHA, we considered all nurses, physicians, and information technology personnel (including those at the VHA Central Office) to be nonadministrative personnel; reclassifying such personnel in the VHA Central Office would likely minimally affect our estimates.

The crosswalk we used imperfectly aligns occupational categories reported in the PAID system and ACS data. Although we corrected some obvious misclassifications, some undoubtedly remain. These likely would not affect our ACS-based comparisons of personnel in clinical sites and not greatly affect our main estimate of overall administrative personnel in the VHA, which used the crosswalked PAID system data only for VHA Central Office personnel. However, the supplementary analysis using crosswalked PAID system data to categorize occupations of all VHA personnel is less secure.

Although more recent data are available, our analyses used 2019 data because staffing patterns in more recent years reflect pandemic-related disruptions and may not be representative of long-term patterns. The VHA’s increasing purchase of private sector care, a trend amplified by the VA Mission Act of 2018, has likely increased administrative costs since then.

Additionally, our data do not speak to whether administrative staffing is excessive in the private sector or inadequate in the VHA. Although quality and cost comparisons do not suggest that the private sector excess yields clinical or cost advantages,^31,32,33,34^ an evaluation of VHA staffing concluded that the program that reimburses private sector health care institutions for veteran care was understaffed.^37^

## Conclusions

In this cross-sectional study, VHA administration appeared leaner than in the private sector, a finding that clashes with the common perception (even our own) that the VHA is excessively bureaucratized. The VHA’s simpler financing scheme and omission of incentives that encourage financial exertions may outweigh administrative inefficiencies due to government regulations and rigidities.

The high administrative costs of US health care have elicited proposals for simplifying payment via single-payer reform,^38^ as well as more modest reforms that would standardize and automate billing.^8,9,11,39^^,^ Over that period, automation has advanced and billing procedures have become more standardized. However, administrative costs have continued to rise,^17,40^ driven partly by an increasingly financialized medical culture.

Implementing uniform credentialing, eligibility determination, and especially payment rates across all payers, as in some other nations, may achieve some administrative savings. However, more fundamental reforms that eliminate per-patient payments and minimize incentives for financial manipulations are likely needed to realize the bulk of potential savings on bureaucracy, savings that could be redirected to improving coverage and care.

## Notes

Supplement 1.
eMethods.
**eTable 1.** Coding Scheme Applied to Assign Detailed Occupations in 2019 American Community Survey to Occupation Groups **eTable 2.** Employment in Private Sector Health Insurers, Brokers, and Related, 2019 **eTable 3.** Full-Time Employment in Clinical Settings, Veterans Health Administration **eTable 4.** Employment in Hospitals, Practitioner Offices, and Other Ambulatory Care Settings **eTable 5.** Employment in Veterans Health Administration and Private Sectors by Occupation Group, Excluding Arizona, New Mexico, Oklahoma, and South Dakota **eTable 6.** Employment in Hospitals, Practitioner Offices, and Other Outpatient Settings by Sector of Employment, Excluding State and Local Government Employees
eReferences.

Supplement 2.
Data Sharing Statement
