# Supplementary material for: Administration’s Share of Personnel in Veterans Health Administration and Private Sector Care
Source: JAMA Netw Open. 2024 Jan 18;7(1):e2352104. doi: 10.1001/jamanetworkopen.2023.52104 (PMC10797450; doi:10.1001/jamanetworkopen.2023.52104)
Supplement: Supplement 1. — eMethods. eTable 1. Coding Scheme Applied to Assign Detailed Occupations in 2019 American Community Survey to Occupation Groups eTable 2. Employment in Private Sector Health Insurers, Brokers, and Related, 2019 eTable 3. Full-Time Employment in Clinical Settings, Veterans Health Administration eTable 4. Employment in Hospitals, Practitioner Offices, and Other Ambulatory Care Settings eTable 5. Employment in Veterans Health Administration and Private Sectors by Occupation Group, Excluding Arizona, New Mexico, Oklahoma, and South Dakota eTable 6. Employment in Hospitals, Practitioner Offices, and Other Outpatient Settings by Sector of Employment, Excluding State and Local Government Employees eReferences. [file jamanetwopen-e2352104-s001.pdf]

Supplemental Online Content

Woolhandler S, Toporek A, Gao J, Moran E, Wilper A, Himmelstein DU. Administration's share of personnel in Veterans Health Administration and private sector care. *JAMA Netw Open*. 2024;7(1):e2352104. doi:10.1001/jamanetworkopen.2023.52104

eMethods.

eTable 1. Coding Scheme Applied to Assign Detailed Occupations in 2019 American Community Survey to Occupation Groups

eTable 2. Employment in Private Sector Health Insurers, Brokers, and Related, 2019

eTable 3. Full-Time Employment in clinical Settings, Veterans Health Administration

eTable 4. Employment in Hospitals, Practitioner Offices, and Other Ambulatory Care Settings

eTable 5. Employment in Veterans Health Administration and Private Sectors by Occupation Group, Excluding Arizona, New Mexico, Oklahoma, and South Dakota

eTable 6. Employment in Hospitals, Practitioner Offices, and Other Outpatient Settings by Sector of Employment, Excluding State And Local Government Employees

eReferences.

This supplemental material has been provided by the authors to give readers additional information about their work.

© 2023 Woolhandler S et al. *JAMA Network Open.*

eMethods

1- Modifications to the EEOC crosswalk of Office of Personnel Management (OPM) occupation codes to Census Occupation Codes[i]

A - Occupation codes that were present in the VHA PAID data but missing from the crosswalk:

OPM code 0593 (insurance accounts) - assigned to Census code 5840 (Insurance claims and policy processing clerks)

OPM code 1160 (financial analyst) - assigned to Census code 0845 (financial and investment analysts)

OPM code 4703 (construction and maintenance supervisors) - assigned to Census code 0440 (managers, other)

B - Occupation codes reclassified based on authors' inspection of crosswalk:

OPM code 0620 (practical nurses) reassigned from Census code 3300 (other technologists and technicians) to 3500 (licensed practical nurses).

OPM code 1035 (public affairs) reassigned from Census code 2805 (broadcast announcers and radio disc jockeys) to 2825 (public relations specialists).

OPM code 1410 (librarians) reassigned from Census code 2400 (archivists, curators, and museum technicians) to 2430 (librarians).

OPM code 1520 (mathematics) reassigned from Census code 1200 (actuaries) to 1210 (mathematicians).

OPM code 1529 (mathematics/statistics) reassigned from Census code 1200 (actuaries) to 1230 (statisticians).

OPM code 1530 (statistics) reassigned from Census code 1200 (actuaries) to 1230 (statisticians).

OPM code 1531 (statistics assistant) reassigned from Census code 1200 (actuaries) to 1240 (other mathematical science occupations).

C – Reclassification of information technology personnel

2/3 of all computer personnel in the VHA's PAID data (including 99% of all computer personnel in the Central Office) were classified under OPM category 2210 (information technology management) which the crosswalk assigns to Census code 0110 (computer and information systems managers). In contrast, in the ACS data, only 8.0% of federal health care IT workers were classified under Census code 0110, and according to the Bureau of Labor Statistics only 9.8% of all IT workers economy wide are classified under Census code 0110. Hence, we assumed that 10% of all VHA IT workers were managers, and assigned the rest to the Information Technology occupation group.

© 2023 Woolhandler S et al. *JAMA Network Open.*

2- Adjustment for the over-estimation of VHA workers in the ACS.

In the ACS, we identified 344,197 federal personnel employed in clinical settings, somewhat more than the 321,643 reported in the PAID system. Therefore, in calculating percentage of administrative personnel in the VHA overall (i.e. combining clinical setting and the VHA Central Office administrative personnel) we first divided 321,643 by 344,197, yielding 0.9345. We then multiplied the ACS figure for administrative personnel by 0.9345, equivalent to a downward adjustment of 6.55%

© 2023 Woolhandler S et al. *JAMA Network Open.*

eTable 1. Coding scheme applied to assign detailed occupations in the 2019 American Community Survey to occupation groups

Census Occupation Code	Occupation Group Assigned
**0010**	Managers and related
**0020**	Managers and related
**0030**	Managers and related
**0040**	Managers and related
**0050**	Managers and related
**0060**	Managers and related
**0100**	Managers and related
**0110**	Managers and related
**0120**	Managers and related
**0130**	Managers and related
**0135**	Managers and related
**0136**	Managers and related
**0137**	Managers and related
**0140**	Managers and related
**0150**	Managers and related
**0160**	Managers and related
**0200**	Occupations not elsewhere classified
**0205**	Managers and related
**0210**	Occupations not elsewhere classified
**0220**	Managers and related
**0230**	Managers and related
**0300**	Managers and related
**0310**	Managers and related
**0320**	Managers and related
**0325**	Managers and related
**0330**	Managers and related
**0335**	Managers and related
**0340**	Managers and related
**0350**	Managers and related
**0360**	Managers and related
**0400**	Managers and related
**0410**	Managers and related
**0420**	Managers and related
**0425**	Managers and related
**0430**	Managers and related
**0500**	Managers and related
**0510**	Managers and related
**0520**	Managers and related

© 2023 Woolhandler S et al. *JAMA Network Open.*

Census Occupation Code	Occupation Group Assigned
**0530**	Managers and related
**0540**	Administrative support, financial
**0560**	Managers and related
**0565**	Managers and related
**0600**	Managers and related
**0620**	Managers and related
**0630**	Managers and related
**0640**	Managers and related
**0650**	Managers and related
**0700**	Professional and technical, except health
**0710**	Managers and related
**0720**	Managers and related
**0725**	Managers and related
**0726**	Managers and related
**0730**	Managers and related
**0735**	Administrative support, except financial
**0740**	Managers and related
**0750**	Managers and related
**0800**	Managers and related
**0810**	Administrative support, except financial
**0820**	Managers and related
**0830**	Managers and related
**0840**	Managers and related
**0850**	Managers and related
**0860**	Managers and related
**0900**	Managers and related
**0910**	Managers and related
**0930**	Managers and related
**0940**	Managers and related
**0950**	Managers and related
**1000**	Information technology
**1005**	Information technology
**1006**	Information technology
**1007**	Information technology
**1010**	Information technology
**1020**	Information technology
**1020**	Information technology
**1021**	Information technology
**1022**	Information technology
**1030**	Information technology

© 2023 Woolhandler S et al. *JAMA Network Open.*

Census Occupation Code	Occupation Group Assigned
**1031**	Information technology
**1032**	Information technology
**1040**	Information technology
**1050**	Information technology
**1060**	Information technology
**1065**	Information technology
**1100**	Information technology
**1105**	Information technology
**1106**	Information technology
**1107**	Information technology
**1108**	Information technology
**1110**	Information technology
**1200**	Managers and related
**1210**	Professional and technical, except health
**1220**	Information technology
**1230**	Professional and technical, except health
**1240**	Professional and technical, except health
**1300**	Professional and technical, except health
**1305**	Professional and technical, except health
**1306**	Professional and technical, except health
**1310**	Occupations not elsewhere classified
**1320**	Professional and technical, except health
**1330**	Professional and technical, except health
**1340**	Professional and technical, except health
**1350**	Professional and technical, except health
**1360**	Professional and technical, except health
**1400**	Information technology
**1410**	Information technology
**1420**	Professional and technical, except health
**1430**	Professional and technical, except health
**1440**	Professional and technical, except health
**1450**	Professional and technical, except health
**1460**	Professional and technical, except health
**1500**	Professional and technical, except health
**1510**	Professional and technical, except health
**1520**	Professional and technical, except health
**1530**	Professional and technical, except health
**1540**	Occupations not elsewhere classified
**1541**	Occupations not elsewhere classified
**1545**	Occupations not elsewhere classified

© 2023 Woolhandler S et al. *JAMA Network Open.*

Census Occupation Code	Occupation Group Assigned
**1550**	Occupations not elsewhere classified
**1551**	Occupations not elsewhere classified
**1555**	Occupations not elsewhere classified
**1560**	Occupations not elsewhere classified
**1600**	Professional and technical, except health
**1610**	Professional and technical, except health
**1640**	Professional and technical, except health
**1650**	Professional and technical, except health
**1660**	Professional and technical, except health
**1700**	Professional and technical, except health
**1710**	Professional and technical, except health
**1720**	Professional and technical, except health
**1740**	Professional and technical, except health
**1745**	Professional and technical, except health
**1750**	Professional and technical, except health
**1760**	Occupations not elsewhere classified
**1800**	Administrative support, financial
**1810**	Professional and technical, except health
**1815**	Professional and technical, except health
**1820**	Social services
**1821**	Social services
**1822**	Social services
**1825**	Social services
**1830**	Professional and technical, except health
**1840**	Occupations not elsewhere classified
**1860**	Professional and technical, except health
**1900**	Food preparation and service
**1910**	Occupations not elsewhere classified
**1920**	Occupations not elsewhere classified
**1930**	Occupations not elsewhere classified
**1930**	Occupations not elsewhere classified
**1935**	Occupations not elsewhere classified
**1950**	Occupations not elsewhere classified
**1960**	Occupations not elsewhere classified
**1965**	Occupations not elsewhere classified
**1980**	Health technologists and technicians
**1970**	Health technologists and technicians
**2000**	Social services
**2001**	Social services
**2002**	Social services

© 2023 Woolhandler S et al. *JAMA Network Open.*

Census Occupation Code	Occupation Group Assigned
**2003**	Social services
**2004**	Social services
**2005**	Social services
**2006**	Social services
**2010**	Social services
**2011**	Social services
**2012**	Social services
**2013**	Social services
**2014**	Social services
**2015**	Social services
**2016**	Social services
**2020**	Social services
**2025**	Social services
**2040**	Social services
**2050**	Managers and related
**2060**	Social services
**2100**	Managers and related
**2105**	Administrative support, except financial
**2110**	Managers and related
**2140**	Administrative support, except financial
**2145**	Administrative support, except financial
**2150**	Administrative support, except financial
**2160**	Administrative support, except financial
**2170**	Administrative support, except financial
**2180**	Administrative support, except financial
**2200**	Professional and technical, except health
**2205**	Social services
**2300**	Social services
**2310**	Social services
**2320**	Social services
**2330**	Social services
**2340**	Social services
**2360**	Social services
**2350**	Social services
**2400**	Administrative support, except financial
**2430**	Professional and technical, except health
**2435**	Professional and technical, except health
**2440**	Administrative support, except financial
**2540**	Social services
**2550**	Social services

© 2023 Woolhandler S et al. *JAMA Network Open.*

Census Occupation Code	Occupation Group Assigned
**2534**	Social services
**2545**	Social services
**2555**	Social services
**2600**	Professional and technical, except health
**2630**	Professional and technical, except health
**2631**	Professional and technical, except health
**2632**	Professional and technical, except health
**2633**	Professional and technical, except health
**2634**	Professional and technical, except health
**2635**	Professional and technical, except health
**2636**	Professional and technical, except health
**2640**	Professional and technical, except health
**2700**	Professional and technical, except health
**2710**	Professional and technical, except health
**2720**	Professional and technical, except health
**2721**	Professional and technical, except health
**2722**	Professional and technical, except health
**2723**	Professional and technical, except health
**2740**	Professional and technical, except health
**2750**	Professional and technical, except health
**2751**	Professional and technical, except health
**2752**	Professional and technical, except health
**2755**	Professional and technical, except health
**2760**	Professional and technical, except health
**2770**	Occupations not elsewhere classified
**2800**	Professional and technical, except health
**2805**	Professional and technical, except health
**2810**	Administrative support, except financial
**2820**	Administrative support, except financial
**2825**	Administrative support, except financial
**2830**	Professional and technical, except health
**2840**	Professional and technical, except health
**2850**	Professional and technical, except health
**2860**	Professional and technical, except health
**2861**	Professional and technical, except health
**2862**	Professional and technical, except health
**2865**	Professional and technical, except health
**2900**	Occupations not elsewhere classified
**2905**	Occupations not elsewhere classified
**2910**	Professional and technical, except health

© 2023 Woolhandler S et al. *JAMA Network Open.*

Census Occupation Code	Occupation Group Assigned
**2920**	Professional and technical, except health
**2960**	Occupations not elsewhere classified
**3000**	Other health diagnosing
**3010**	Other health diagnosing
**3030**	Other health assessment and treating
**3040**	Other health diagnosing
**3050**	Other health assessment and treating
**3060**	Physicians
**3090**	Physicians
**3100**	Physicians
**3110**	Other health assessment and treating
**3120**	Other health diagnosing
**3130**	Registered nurses
**3140**	Therapists
**3150**	Therapists
**3160**	Therapists
**3200**	Health technologists and technicians
**3210**	Therapists
**3220**	Therapists
**3230**	Therapists
**3235**	Therapists
**3240**	Therapists
**3245**	Therapists
**3250**	Other health diagnosing
**3255**	Registered nurses
**3256**	Registered nurses
**3257**	Registered nurses
**3258**	Registered nurses
**3260**	Other health diagnosing
**3261**	Registered nurses
**3270**	Other health diagnosing
**3300**	Health technologists and technicians
**3310**	Health technologists and technicians
**3320**	Health technologists and technicians
**3321**	Health technologists and technicians
**3322**	Health technologists and technicians
**3323**	Health technologists and technicians
**3324**	Health technologists and technicians
**3330**	Health technologists and technicians
**3400**	Health technologists and technicians

© 2023 Woolhandler S et al. *JAMA Network Open.*

Census Occupation Code	Occupation Group Assigned
**3401**	Health technologists and technicians
**3402**	Health technologists and technicians
**3410**	Health technologists and technicians
**3420**	Health technologists and technicians
**3421**	Health technologists and technicians
**3422**	Health technologists and technicians
**3423**	Health technologists and technicians
**3424**	Health technologists and technicians
**3500**	Licensed practical nurses
**3510**	Administrative support, except financial
**3515**	Administrative support, except financial
**3520**	Occupations not elsewhere classified
**3530**	Health technologists and technicians
**3535**	Health technologists and technicians
**3540**	Health technologists and technicians
**3540**	Health technologists and technicians
**3545**	Health technologists and technicians
**3550**	Health technologists and technicians
**3570**	Health technologists and technicians
**3600**	Other health service
**3603**	Other health service
**3601**	Other health service
**3602**	Other health service
**3604**	Other health service
**3605**	Other health service
**3610**	Other health service
**3620**	Other health service
**3630**	Occupations not elsewhere classified
**3640**	Other health service
**3645**	Other health service
**3646**	Administrative support, except financial
**3647**	Other health service
**3648**	Other health service
**3649**	Other health service
**3650**	Other health service
**3655**	Other health service
**3700**	Managers and related
**3710**	Managers and related
**3720**	Managers and related
**3725**	Managers and related

© 2023 Woolhandler S et al. *JAMA Network Open.*

Census Occupation Code	Occupation Group Assigned
**3730**	Managers and related
**3740**	Occupations not elsewhere classified
**3750**	Occupations not elsewhere classified
**3800**	Occupations not elsewhere classified
**3801**	Occupations not elsewhere classified
**3802**	Occupations not elsewhere classified
**3820**	Occupations not elsewhere classified
**3830**	Occupations not elsewhere classified
**3840**	Occupations not elsewhere classified
**3850**	Occupations not elsewhere classified
**3860**	Occupations not elsewhere classified
**3870**	Occupations not elsewhere classified
**3900**	Occupations not elsewhere classified
**3910**	Occupations not elsewhere classified
**3920**	Occupations not elsewhere classified
**3930**	Occupations not elsewhere classified
**3940**	Occupations not elsewhere classified
**3945**	Occupations not elsewhere classified
**3946**	Occupations not elsewhere classified
**3950**	Occupations not elsewhere classified
**3955**	Occupations not elsewhere classified
**3960**	Occupations not elsewhere classified
**4000**	Food preparation and service
**4010**	Managers and related
**4020**	Food preparation and service
**4030**	Food preparation and service
**4040**	Food preparation and service
**4050**	Food preparation and service
**4055**	Food preparation and service
**4060**	Food preparation and service
**4110**	Food preparation and service
**4120**	Food preparation and service
**4130**	Food preparation and service
**4140**	Food preparation and service
**4150**	Food preparation and service
**4160**	Food preparation and service
**4200**	Managers and related
**4210**	Managers and related
**4220**	Cleaning, building service and laundry
**4230**	Cleaning, building service and laundry

© 2023 Woolhandler S et al. *JAMA Network Open.*

Census Occupation Code	Occupation Group Assigned
**4240**	Cleaning, building service and laundry
**4250**	Occupations not elsewhere classified
**4251**	Occupations not elsewhere classified
**4252**	Occupations not elsewhere classified
**4255**	Occupations not elsewhere classified
**4300**	Managers and related
**4320**	Managers and related
**4330**	Managers and related
**4340**	Professional and technical, except health
**4350**	Occupations not elsewhere classified
**4400**	Occupations not elsewhere classified
**4410**	Professional and technical, except health
**4420**	Occupations not elsewhere classified
**4430**	Occupations not elsewhere classified
**4435**	Occupations not elsewhere classified
**4460**	Occupations not elsewhere classified
**4461**	Occupations not elsewhere classified
**4465**	Occupations not elsewhere classified
**4500**	Occupations not elsewhere classified
**4510**	Occupations not elsewhere classified
**4520**	Occupations not elsewhere classified
**4521**	Occupations not elsewhere classified
**4522**	Occupations not elsewhere classified
**4525**	Occupations not elsewhere classified
**4530**	Occupations not elsewhere classified
**4540**	Occupations not elsewhere classified
**4550**	Occupations not elsewhere classified
**4600**	Occupations not elsewhere classified
**4610**	Other health service
**4620**	Social services
**4621**	Social services
**4622**	Social services
**4640**	Occupations not elsewhere classified
**4650**	Occupations not elsewhere classified
**4655**	Other health service
**4700**	Managers and related
**4710**	Managers and related
**4720**	Occupations not elsewhere classified
**4740**	Occupations not elsewhere classified
**4750**	Occupations not elsewhere classified

© 2023 Woolhandler S et al. *JAMA Network Open.*

Census Occupation Code	Occupation Group Assigned
**4760**	Occupations not elsewhere classified
**4800**	Administrative support, except financial
**4810**	Administrative support, financial
**4820**	Administrative support, financial
**4830**	Administrative support, except financial
**4840**	Occupations not elsewhere classified
**4850**	Occupations not elsewhere classified
**4900**	Occupations not elsewhere classified
**4920**	Administrative support, except financial
**4930**	Occupations not elsewhere classified
**4940**	Administrative support, except financial
**4950**	Occupations not elsewhere classified
**4960**	Occupations not elsewhere classified
**4965**	Occupations not elsewhere classified
**5000**	Managers and related
**5010**	Administrative support, except financial
**5020**	Administrative support, except financial
**5030**	Administrative support, except financial
**5040**	Administrative support, except financial
**5100**	Administrative support, financial
**5110**	Administrative support, financial
**5120**	Administrative support, financial
**5130**	Occupations not elsewhere classified
**5140**	Administrative support, financial
**5150**	Administrative support, except financial
**5160**	Administrative support, financial
**5165**	Administrative support, financial
**5200**	Administrative support, financial
**5210**	Administrative support, except financial
**5220**	Administrative support, except financial
**5230**	Administrative support, financial
**5240**	Administrative support, financial
**5250**	Administrative support, financial
**5260**	Administrative support, except financial
**5300**	Administrative support, except financial
**5310**	Administrative support, except financial
**5320**	Administrative support, except financial
**5330**	Administrative support, financial
**5340**	Administrative support, financial
**5350**	Administrative support, except financial

© 2023 Woolhandler S et al. *JAMA Network Open.*

Census Occupation Code	Occupation Group Assigned
**5360**	Administrative support, except financial
**5400**	Administrative support, except financial
**5410**	Administrative support, except financial
**5420**	Administrative support, except financial
**5500**	Administrative support, except financial
**5510**	Administrative support, except financial
**5520**	Administrative support, except financial
**5521**	Administrative support, except financial
**5522**	Administrative support, except financial
**5530**	Occupations not elsewhere classified
**5540**	Administrative support, except financial
**5550**	Occupations not elsewhere classified
**5560**	Administrative support, except financial
**5600**	Administrative support, except financial
**5610**	Administrative support, except financial
**5620**	Occupations not elsewhere classified
**5630**	Administrative support, except financial
**5700**	Administrative support, except financial
**5740**	Administrative support, except financial
**5730**	Administrative support, except financial
**5720**	Administrative support, except financial
**5710**	Administrative support, except financial
**5800**	Information technology
**5810**	Administrative support, except financial
**5820**	Administrative support, except financial
**5830**	Administrative support, except financial
**5840**	Administrative support, financial
**5850**	Administrative support, except financial
**5860**	Administrative support, except financial
**5900**	Administrative support, except financial
**5910**	Administrative support, except financial
**5920**	Administrative support, except financial
**5930**	Administrative support, except financial
**5940**	Administrative support, except financial
**6000**	Managers and related
**6005**	Managers and related
**6010**	Occupations not elsewhere classified
**6020**	Occupations not elsewhere classified
**6040**	Occupations not elsewhere classified
**6050**	Occupations not elsewhere classified

© 2023 Woolhandler S et al. *JAMA Network Open.*

Census Occupation Code	Occupation Group Assigned
**6100**	Occupations not elsewhere classified
**6110**	Occupations not elsewhere classified
**6120**	Occupations not elsewhere classified
**6130**	Occupations not elsewhere classified
**6200**	Managers and related
**6210**	Occupations not elsewhere classified
**6220**	Building construction and maintenance
**6230**	Building construction and maintenance
**6240**	Building construction and maintenance
**6250**	Building construction and maintenance
**6260**	Occupations not elsewhere classified
**6300**	Building construction and maintenance
**6305**	Building construction and maintenance
**6310**	Building construction and maintenance
**6320**	Occupations not elsewhere classified
**6330**	Building construction and maintenance
**6350**	Building construction and maintenance
**6355**	Building construction and maintenance
**6360**	Occupations not elsewhere classified
**6400**	Building construction and maintenance
**6410**	Building construction and maintenance
**6420**	Building construction and maintenance
**6430**	Building construction and maintenance
**6440**	Building construction and maintenance
**6441**	Building construction and maintenance
**6442**	Building construction and maintenance
**6460**	Building construction and maintenance
**6500**	Building construction and maintenance
**6510**	Building construction and maintenance
**6515**	Building construction and maintenance
**6520**	Occupations not elsewhere classified
**6530**	Building construction and maintenance
**6540**	Occupations not elsewhere classified
**6600**	Building construction and maintenance
**6660**	Managers and related
**6700**	Building construction and maintenance
**6710**	Building construction and maintenance
**6720**	Building construction and maintenance
**6730**	Occupations not elsewhere classified
**6740**	Occupations not elsewhere classified

© 2023 Woolhandler S et al. *JAMA Network Open.*

Census Occupation Code	Occupation Group Assigned
**6750**	Occupations not elsewhere classified
**6760**	Building construction and maintenance
**6765**	Building construction and maintenance
**6800**	Occupations not elsewhere classified
**6820**	Occupations not elsewhere classified
**6830**	Building construction and maintenance
**6835**	Occupations not elsewhere classified
**6840**	Occupations not elsewhere classified
**6850**	Occupations not elsewhere classified
**6910**	Occupations not elsewhere classified
**6920**	Occupations not elsewhere classified
**6930**	Occupations not elsewhere classified
**6940**	Occupations not elsewhere classified
**6950**	Occupations not elsewhere classified
**7000**	Managers and related
**7010**	Information technology
**7020**	Building construction and maintenance
**7030**	Building construction and maintenance
**7040**	Building construction and maintenance
**7050**	Building construction and maintenance
**7100**	Building construction and maintenance
**7110**	Building construction and maintenance
**7120**	Building construction and maintenance
**7130**	Building construction and maintenance
**7140**	Occupations not elsewhere classified
**7150**	Occupations not elsewhere classified
**7160**	Occupations not elsewhere classified
**7200**	Occupations not elsewhere classified
**7210**	Occupations not elsewhere classified
**7220**	Occupations not elsewhere classified
**7240**	Occupations not elsewhere classified
**7260**	Occupations not elsewhere classified
**7300**	Building construction and maintenance
**7310**	Building construction and maintenance
**7315**	Building construction and maintenance
**7320**	Building construction and maintenance
**7330**	Occupations not elsewhere classified
**7340**	Building construction and maintenance
**7350**	Building construction and maintenance
**7360**	Building construction and maintenance

© 2023 Woolhandler S et al. *JAMA Network Open.*

Census Occupation Code	Occupation Group Assigned
**7410**	Building construction and maintenance
**7420**	Building construction and maintenance
**7430**	Building construction and maintenance
**7440**	Occupations not elsewhere classified
**7510**	Occupations not elsewhere classified
**7520**	Professional and technical, except health
**7540**	Building construction and maintenance
**7550**	Building construction and maintenance
**7560**	Building construction and maintenance
**7600**	Building construction and maintenance
**7610**	Occupations not elsewhere classified
**7620**	Building construction and maintenance
**7630**	Building construction and maintenance
**7640**	Building construction and maintenance
**7700**	Managers and related
**7710**	Occupations not elsewhere classified
**7720**	Occupations not elsewhere classified
**7730**	Occupations not elsewhere classified
**7740**	Occupations not elsewhere classified
**7750**	Occupations not elsewhere classified
**7800**	Food preparation and service
**7810**	Food preparation and service
**7830**	Food preparation and service
**7840**	Food preparation and service
**7850**	Food preparation and service
**7855**	Food preparation and service
**7900**	Information technology
**7905**	Occupations not elsewhere classified
**7920**	Occupations not elsewhere classified
**7925**	Occupations not elsewhere classified
**7930**	Occupations not elsewhere classified
**7940**	Occupations not elsewhere classified
**7950**	Occupations not elsewhere classified
**7960**	Occupations not elsewhere classified
**8000**	Occupations not elsewhere classified
**8010**	Occupations not elsewhere classified
**8020**	Occupations not elsewhere classified
**8025**	Occupations not elsewhere classified
**8020**	Occupations not elsewhere classified
**8030**	Occupations not elsewhere classified

© 2023 Woolhandler S et al. *JAMA Network Open.*

Census Occupation Code	Occupation Group Assigned
**8040**	Occupations not elsewhere classified
**8060**	Occupations not elsewhere classified
**8100**	Occupations not elsewhere classified
**8120**	Occupations not elsewhere classified
**8130**	Occupations not elsewhere classified
**8140**	Occupations not elsewhere classified
**8150**	Occupations not elsewhere classified
**8160**	Building construction and maintenance
**8200**	Occupations not elsewhere classified
**8210**	Building construction and maintenance
**8220**	Occupations not elsewhere classified
**8225**	Occupations not elsewhere classified
**8230**	Occupations not elsewhere classified
**8240**	Occupations not elsewhere classified
**8250**	Occupations not elsewhere classified
**8255**	Occupations not elsewhere classified
**8256**	Occupations not elsewhere classified
**8260**	Occupations not elsewhere classified
**8300**	Cleaning, building service and laundry
**8310**	Cleaning, building service and laundry
**8320**	Occupations not elsewhere classified
**8330**	Occupations not elsewhere classified
**8335**	Occupations not elsewhere classified
**8340**	Occupations not elsewhere classified
**8350**	Occupations not elsewhere classified
**8360**	Occupations not elsewhere classified
**8365**	Occupations not elsewhere classified
**8400**	Occupations not elsewhere classified
**8410**	Occupations not elsewhere classified
**8420**	Occupations not elsewhere classified
**8430**	Occupations not elsewhere classified
**8440**	Occupations not elsewhere classified
**8450**	Occupations not elsewhere classified
**8460**	Occupations not elsewhere classified
**8465**	Occupations not elsewhere classified
**8500**	Occupations not elsewhere classified
**8510**	Building construction and maintenance
**8520**	Occupations not elsewhere classified
**8530**	Occupations not elsewhere classified
**8540**	Occupations not elsewhere classified

© 2023 Woolhandler S et al. *JAMA Network Open.*

Census Occupation Code	Occupation Group Assigned
**8550**	Occupations not elsewhere classified
**8555**	Building construction and maintenance
**8600**	Building construction and maintenance
**8610**	Building construction and maintenance
**8620**	Building construction and maintenance
**8630**	Building construction and maintenance
**8640**	Occupations not elsewhere classified
**8650**	Occupations not elsewhere classified
**8710**	Occupations not elsewhere classified
**8720**	Occupations not elsewhere classified
**8730**	Occupations not elsewhere classified
**8740**	Occupations not elsewhere classified
**8750**	Occupations not elsewhere classified
**8760**	Health technologists and technicians
**8800**	Occupations not elsewhere classified
**8810**	Occupations not elsewhere classified
**8830**	Occupations not elsewhere classified
**8840**	Occupations not elsewhere classified
**8850**	Occupations not elsewhere classified
**8860**	Occupations not elsewhere classified
**8900**	Occupations not elsewhere classified
**8910**	Occupations not elsewhere classified
**8920**	Occupations not elsewhere classified
**8930**	Occupations not elsewhere classified
**8940**	Occupations not elsewhere classified
**8950**	Occupations not elsewhere classified
**8960**	Occupations not elsewhere classified
**8965**	Occupations not elsewhere classified
**8990**	Occupations not elsewhere classified
**9000**	Managers and related
**9005**	Managers and related
**9030**	Occupations not elsewhere classified
**9040**	Occupations not elsewhere classified
**9050**	Occupations not elsewhere classified
**9110**	Other health service
**9120**	Occupations not elsewhere classified
**9121**	Occupations not elsewhere classified
**9122**	Occupations not elsewhere classified
**9130**	Occupations not elsewhere classified
**9140**	Occupations not elsewhere classified

© 2023 Woolhandler S et al. *JAMA Network Open.*

Census Occupation Code	Occupation Group Assigned
**9141**	Occupations not elsewhere classified
**9142**	Occupations not elsewhere classified
**9150**	Occupations not elsewhere classified
**9200**	Occupations not elsewhere classified
**9230**	Occupations not elsewhere classified
**9240**	Occupations not elsewhere classified
**9260**	Occupations not elsewhere classified
**9300**	Occupations not elsewhere classified
**9310**	Occupations not elsewhere classified
**9330**	Occupations not elsewhere classified
**9340**	Occupations not elsewhere classified
**9350**	Occupations not elsewhere classified
**9360**	Occupations not elsewhere classified
**9365**	Occupations not elsewhere classified
**9410**	Occupations not elsewhere classified
**9415**	Occupations not elsewhere classified
**9420**	Occupations not elsewhere classified
**9430**	Occupations not elsewhere classified
**9500**	Occupations not elsewhere classified
**9510**	Occupations not elsewhere classified
**9520**	Occupations not elsewhere classified
**9560**	Occupations not elsewhere classified
**9570**	Occupations not elsewhere classified
**9600**	Occupations not elsewhere classified
**9610**	Occupations not elsewhere classified
**9620**	Occupations not elsewhere classified
**9630**	Occupations not elsewhere classified
**9640**	Occupations not elsewhere classified
**9645**	Occupations not elsewhere classified
**9650**	Occupations not elsewhere classified
**9720**	Occupations not elsewhere classified
**9730**	Occupations not elsewhere classified
**9740**	Occupations not elsewhere classified
**9750**	Occupations not elsewhere classified
**9760**	Occupations not elsewhere classified

© 2023 Woolhandler S et al. *JAMA Network Open.*

eTable 2. Employment in private (non-VHA) sector health insurers, brokers and related, 2019

**Insurance Industry Category**	**Total # of Employees**	**Share of Employees Attributable to Health Insurance***	**# of Employees Attributable to Health Insurance (thousands)**
Direct health and medical Insurance carriers	573,300	1.00	573,300
Insurance agencies and brokerages	1,191,700	.3587	427,500
**Total health-insurance personnel**			**1,000,800**

Source: Bureau of Labor Statistics. Employment Projections. National Employment Matrix. Employment by industry, occupation, and percent distribution, 2019 and projected 2029. Note that BLS does not report confidence intervals on its estimates.

*Percent of employees in insurance agencies and brokers, and in other insurance related activities was calculated based on the share of all insurance carrier employees attributable to "direct health and medical insurance carriers".

© 2023 Woolhandler S et al. *JAMA Network Open.*

eTable 3. Employment in clinical settings, Veterans Health Administration (VHA). 2019 PAID data cross-walked to Census Occupation Codes

	Percent of Total FTEs (N= 321,643)
Managers	7.1
Administrative support, non-financial	12.9
Administrative support, financial	2.0
**Total administration**	**21.9**
Professional/technical except health (e.g. engineers)	1.6
Social Services	7.2
Physicians	6.9
Other health diagnosing (e.g. dentists)	0.9
Registered nurses	23.6
Therapists	2.1
Other health assessing/treating (e.g. pharmacists)	4.2
Health technologists/technicians	6.3
Licensed practical nurses	4.5
Other health services, e.g. aides	4.6
Food services	2.8
Cleaning/building services/laundry	3.7
Building construction/maintenance	1.1
Information technology	1.0
Occupations not elsewhere classified	6.9

© 2023 Woolhandler S et al. *JAMA Network Open.*

eTable 4. Employment in hospitals, practitioners' offices and other ambulatory care settings, Veterans Health Administration (VHA) vs. private (non-VHA) sectors based on calculated FTEs, 2019 American Community Survey

	**Veterans Health Administration**		**Private Sector**	
	Percent of Total	95% CI	Percent of Total	95% CI
Managers	10.6	8.3 - 12.8	9.0	5.6 - 12.4
Administrative support, non-financial	7.8	6.5 - 9.0	11.6	9.2 - 14.0
Administrative support, financial	1.7	1.1 - 2.4	3.1	2.8 - 3.3
**Total administration**	**20.1**	**17.9 - 22.2**	**23.6**	**22.8-24.5**
Professional/technical except health (e.g. engineers)	1.3	0.8 - 1.8	0.8	0.7 - 0.9
Social Services	6.3	5.4 - 7.3	4.7	3.8 - 5.5
Physicians	8.3	3.5 - 13.2	8.4	0.7 - 16.1
Other health diagnosing (e.g. dentists)	1.3	0.7 - 1.9	2.4	2.2 - 2.5
Registered nurses	23.2	20.4 - 26.1	20.6	18.2 - 23.0
Therapists	2.4	1.7 - 3.1	4.4	3.2 - 5.6
Other health assessing/treating (e.g. pharmacists)	4.4	3.5 - 5.2	2.2	2.0 - 2.4
Health technologists/technicians	9.0	7.4 - 10.6	10.2	9.1 - 11.2
Licensed practical nurses	3.1	2.4 - 3.9	2.3	2.1 - 2.5
Other health services, e.g. aides	9.4	7.6 - 11.2	12.5	9.2 - 15.9
Food services	1.3	0.7 - 1.8	0.8	0.5 - 1.0
Cleaning/building services/laundry	2.7	1.8 - 3.5	2.0	1.7 - 2.3
Building construction/maintenance	2.2	1.7 - 2.7	0.8	0.6 - 1.0
Information technology	1.6	1.1 - 2.1	1.7	1.2 - 2.2
Occupations not elsewhere classified	3.3	2.4 - 4.2	2.7	2.5 - 2.9

Note: Excludes nursing homes, home care, and "other health care services", as well personnel in health insurers and brokers, revenue cycle management firms, central offices of the VA and civilian sector providers, and organizations providing services or supplies to health care providers, and all employees of state or local governments.

Note: Excludes military personnel and persons with a job but not at work.

© 2023 Woolhandler S et al. *JAMA Network Open.*

eTable 5. Employment in the VHA and private (non-VHA) sectors according to occupation groups, excluding persons living in Arizona, New Mexico, Oklahoma, and South Dakota (states accounting for 75.9% of Indian Health Service employment), 2019 American Community Survey

	Veterans Health Administration (weighted N = 316,789)		Private Sector (weighted N= 11,677,584	
	% of workforce	95% CI	% of workforce	95% CI
Managers	10.1	8.6 - 11.7	8.2	8.0 - 8.4
Admin Support, non-financial	7.5	6.3 - 8.6	12.2	12.0 - 12.5
Admin support, financial	1.7	1.0 - 2.3	3.1	2.9 - 3.2
**Total Administration**	**19.3**	**17.5 - 21.0**	**23.4**	**23.1 - 23.8**
Professional/technical except health (e.g. engineers)	1.3	0.9 - 1.8	0.8	0.7 - 0.9
Social Services	6.1	5.2 - 7.0	4.9	4.8 - 5.1
Physicians	7.4	6.3 - 8.6	6.5	6.3 - 6.7
Other health diagnosing (e.g. dentists)	1.2	0.7 - 1.7	2.4	2.3 - 2.5
Registered nurses	24.1	21.9 - 26.3	21.2	20.9 - 21.6
Therapists	2.6	2.0 - 3.2	4.7	4.5 - 4.8
Other health assessing/treating (e.g. pharmacists)	4.5	3.8 - 5.3	2.2	2.1 - 2.2
Health technologists/technicians	9.1	7.8 - 10.4	10.4	10.2 - 10.6
Licensed practical nurses	3.2	2.4 - 4.0	2.4	2.2 - 2.5
Other health services, e.g. aides	9.8	8.6 - 11.0	13.3	13.1 - 13.6
Food services	1.3	0.8 - 1.9	0.8	0.8 - 0.9
Cleaning/building services/laundry	2.8	2.1 - 3.6	2.1	2.0 - 2.2
Building construction/maintenance	2.1	1.6 - 2.6	0.7	0.7 - 0.8
Information technology	1.6	1.1 - 2.1	1.6	1.5 - 1.7
Occupations not elsewhere classified	3.5	2.7 - 4.3	2.6	2.5 - 2.8

Note: Excludes nursing homes, home care, and “other health care services”, as well personnel in health insurers and brokers, revenue cycle management firms, central offices of the VA and civilian sector providers, and organizations providing services or supplies to health care providers, and all employees of state or local governments.

Note: Excludes military personnel and persons with a job but not at work.

© 2023 Woolhandler S et al. *JAMA Network Open.*

eTable 6. Employment in hospitals, practitioners' offices and other outpatient settings, according to sector of employment, excluding state and local government employees, 2019 American Community Survey

	Veterans Health Administration		Private Sector (Excluding State/Local Government Employees)	
	Percent of Personnel	95% CI	Percent of Personnel	95% CI
Managers	10.1	8.7 - 11.6	8.1	7.9 - 8.3
Administrative support, non-financial	7.9	6.7 - 9.1	12.4	12.1 - 12.7
Administrative support, financial	1.7	1.1 - 2.3	3.1	3.0 - 3.3
**Total Administration**	**19.8**	**18.1 - 21.4**	**23.6**	**23.3 - 24.0**
Professional/technical except health (e.g. engineers)	1.3	0.9 - 1.8	0.8	0.7 - 0.8
Social Services	6.3	5.4 - 7.1	4.7	4.6 - 4.9
Physicians	7.2	6.1 - 8.2	6.4	6.2 - 6.6
Other health diagnosing (e.g. dentists)	1.3	0.7 - 1.8	2.5	2.4 - 2.6
Registered nurses	23.7	21.6 - 25.8	21.1	20.8 - 21.5
Therapists	2.5	2.0 - 3.1	4.8	4.6 - 5.0
Other health assessing/treating (e.g. pharmacists)	4.4	3.7 - 5.2	2.2	2.1 - 2.2
Health technologists/technicians	9.3	8.1 - 10.4	10.4	10.2 - 10.7
Licensed practical nurses	3.2	2.4 - 3.9	2.3	2.2 - 2.4
Other health services, e.g. aides	9.8	8.6 - 11.0	13.5	13.2 - 13.7
Food services	1.3	0.8 - 1.8	0.8	0.7 - 0.9
Cleaning/building services/laundry	2.8	2.0 - 3.5	2.0	1.9 - 2.1
Building construction/maintenance	2.2	1.7 - 2.8	0.7	0.6 - 0.8
Information technology	1.6	1.2 - 2.1	1.5	1.4 - 1.6
Occupations not elsewhere classified	3.4	2.7 - 4.1	2.6	2.5 - 2.7

© 2023 Woolhandler S et al. *JAMA Network Open.*

Note: Excludes nursing homes, home care, and "other health care services", as well personnel in health insurers and brokers, revenue cycle management firms, central offices of the VA and civilian sector providers, and organizations providing services or supplies to health care providers, and all employees of state or local governments.

Note: Excludes military personnel and persons with a job but not at work.

© 2023 Woolhandler S et al. *JAMA Network Open.*

eReferences

[i] EEOC Federal Sector Occupation Cross-Classification Table (December 20, 2021). https://www.eeoc.gov/federal-sector/management-directive/eeoc-federal-sector-occupation-cross-classification-table (accessed June 8, 2023).
