# Supplementary material for: Administration’s Share of Personnel in Veterans Health Administration and Private Sector Care
Source: JAMA Netw Open. 2024 Jan 18;7(1):e2352104. doi: 10.1001/jamanetworkopen.2023.52104 (PMC10797450; doi:10.1001/jamanetworkopen.2023.52104)
Supplement: Supplement 2. — Data Sharing Statement [file jamanetwopen-e2352104-s002.pdf]

Data Sharing Statement

Woolhandler. Administration's Share of Personnel in Veterans Health Administration and Private Sector Care. *JAMA Netw Open*. Published online January 18, 2024. doi:10.1001/jamanetworkopen.2023.52104

Data
Data available: No

Additional Information
Explanation for why data not available: The VHA personnel data used as part of the analysis is confidential
